# Morphological-anatomical and chemical features of *Copernicia alba* fruits and seeds, a palm from Brazilian Pantanal

**DOI:** 10.1371/journal.pone.0282775

**Published:** 2023-03-10

**Authors:** Rosa Helena da Silva, Anny Beatriz Silva, Rafael Arruda, Isabela Pavão Vargas, Fabiana Fonseca Zanoelo, Geraldo Alves Damasceno Junior, Arnildo Pott, Rosani do Carmo de Oliveira Arruda

**Affiliations:** 1 Laboratório de Ecologia Vegetal, Instituto de Biociências, Universidade Federal de Mato Grosso do Sul, Campo Grande, Mato Grosso do Sul, Brasil; 2 Laboratório de Anatomia Vegetal, Instituto de Biociências, Universidade Federal de Mato Grosso do Sul, Campo Grande, Mato Grosso do Sul, Brasil; 3 Laboratório de Quiropterologia Neotropical, Instituto de Ciências Naturais, Humanas e Sociais, Universidade Federal de Mato Grosso, Sinop, Mato Grosso, Brasil; 4 Laboratório de Bioquímica Geral e Microrganismos, Instituto de Biociências, Universidade Federal de Mato Grosso do Sul, Campo Grande, Mato Grosso do Sul, Brasil; 5 Programa de Pós-Graduação em Biologia Vegetal, Instituto de Biociências, Universidade Federal de Mato Grosso do Sul, Campo Grande, Mato Grosso do Sul, Brasil; Universidade Federal de Uberlandia - Campus Umuarama, BRAZIL

## Abstract

*Copernicia alba* (Arecaceae) is a palm tree regionally known as *carandá* that forms large populations and produces abundant fruits, an important food source for the local fauna in Brazilian wetlands. The fruits present morphological variations regarding color, shape and dimensions. In this study fruits of different shapes were collected and processed following routine techniques in plant morphology, and biochemistry analysis of endosperm. (hemicellulose) The fruits isdark, of the berry type, with partially fibrous pericarp, rich in phenolic compounds; the ruminated seed coat also contains phenols; the endosperm, formed of cells with highly thickened, not lignified hemicellulosic walls, stores xyloses, proteins and lipids. The embryo is short and straight. Xylose is the leading sugar of xylan, which can be liberated by hydrolysis with specific enzymes, such as xylanases. This sugar is of interest in several industrial sectors, such as the production of biofuels and xylitol for foods. Excepting depth of seed rumination, *C*. *alba* fruits do not have relevant differences in anatomy and classes of substances detected. The fruit yield showed differences associated with its shape, indicating the best utilization. Considering fruit anatomical features and tissue composition, we highlight that the seeds of *C*. *alba* have the potential as a new functional food source.

## Introduction

Palms are one of the most economically important families of plants to man, and, in the tropics, they have equal importance to legumes being crops of coconut (*Cocos nucifera* L.), oil palm (*Elaeis oleifera* (Kunth) Cortés),) and date palm (*Phoenix dactylifera* L.) are well-known, providing numerous useful valuable products such as foodstuffs, fibers, and medicine [1]. In tropical regions that concentrate 90% of Arecaceae diversity, the reproductive parts are generally difficult to access; on the other hand, they stay longer on the mother plant due to their fruit or seed hardness and can be easily found, so becoming a key structure in species identification in the field [2, 3].

The South American Pantanal is the most significant tropical continuous floodplain in the world [4], with many species of valuable plants that can serve as food, some of them being monodominant in their landscapes. Among these monodominant species in the Pantanal landscape, we can highlight the palms *Attlalea phalerata* Mart. Ex Spreng., *Mauritia flexuosa* L.f., *Attalea speciosa* Mart. Ex Spreng. and *Copernicia alba* Morong ex Morong & Britton [4]. These species are still poorly studied and have an excellent potential to be incorporated into the bioeconomy.

*Copernicia alba* is one of these palms, native to South America, and is found in Brazil, northern Argentina, Paraguay, and southern Bolivia [5–7]. In Brazil, this species is regionally known as *carandá* and is widely distributed in wetlands, in large monodominant formations along the Paraguay River known as carandazais [8]. The estimated *carandá* area in the Pantanal vegetation is 2.3%, the second most representative monotypic formation [9].

The carandá generally, occur in open areas with poorly drained soils, indicative of periodically flooded soils with high pH (greater than 5.7) and high amounts of magnesium [4, 5]. The spherical canopy is distinct in the landscape since the leaves are a characteristic pattern that gives the plant ornamental potential, like others of the Coryphoideae subfamily [3].) The staminate flowers are at the base, and the pistillate is on the top of the inflorescence branches. Fruit development begins between March and April, with unripe fruits, showing the peak of mature fruits in May [6, 10] estimated an average annual production of 23.25 ton.ha-¹ of fruits and 5.3 ton.ha-¹ of seeds. Despite the potential uses of *Copernicia alba* more study is needed on their morpho-anatomical and chemical structure that supports their utilization.

Considering the possible importance of *Copernicia alba* fruit as food or even as non-food product it is essential to study its chemical composition. One promising component to study is hemicellulose. Hemicellulose is a highly heterogeneous polysaccharide of low molecular mass, composed mainly of pentoses, hexoses, and uronic acids [11]. It can present different compositions depending on the plant species, development stage, and tissue. As the main sugar, it can be classified as xylans, mannans, arabinoxylans, arabinogalactans, and arabinans [12]. Xylan is the most abundant hemicellulose, composed of β-1,4- D-linked xylose residues chained by glycosidic bonds, and is a major structural component of plant cell walls. This polymer’s enzymes, among which endo-xylanases (EC 3.2.1.8) play a key role [13, 14]. Xylan-derived sugar can be converted to value-added products such as xylitol, used as an additive in food, cosmetics, and medicine [12]. Furthermore, it can be used as an excellent alternative to fossil fuels in second-generation bioethanol production through the fermentation of sugars derived from lignocellulosic biomass [15].

**Fig 1:**
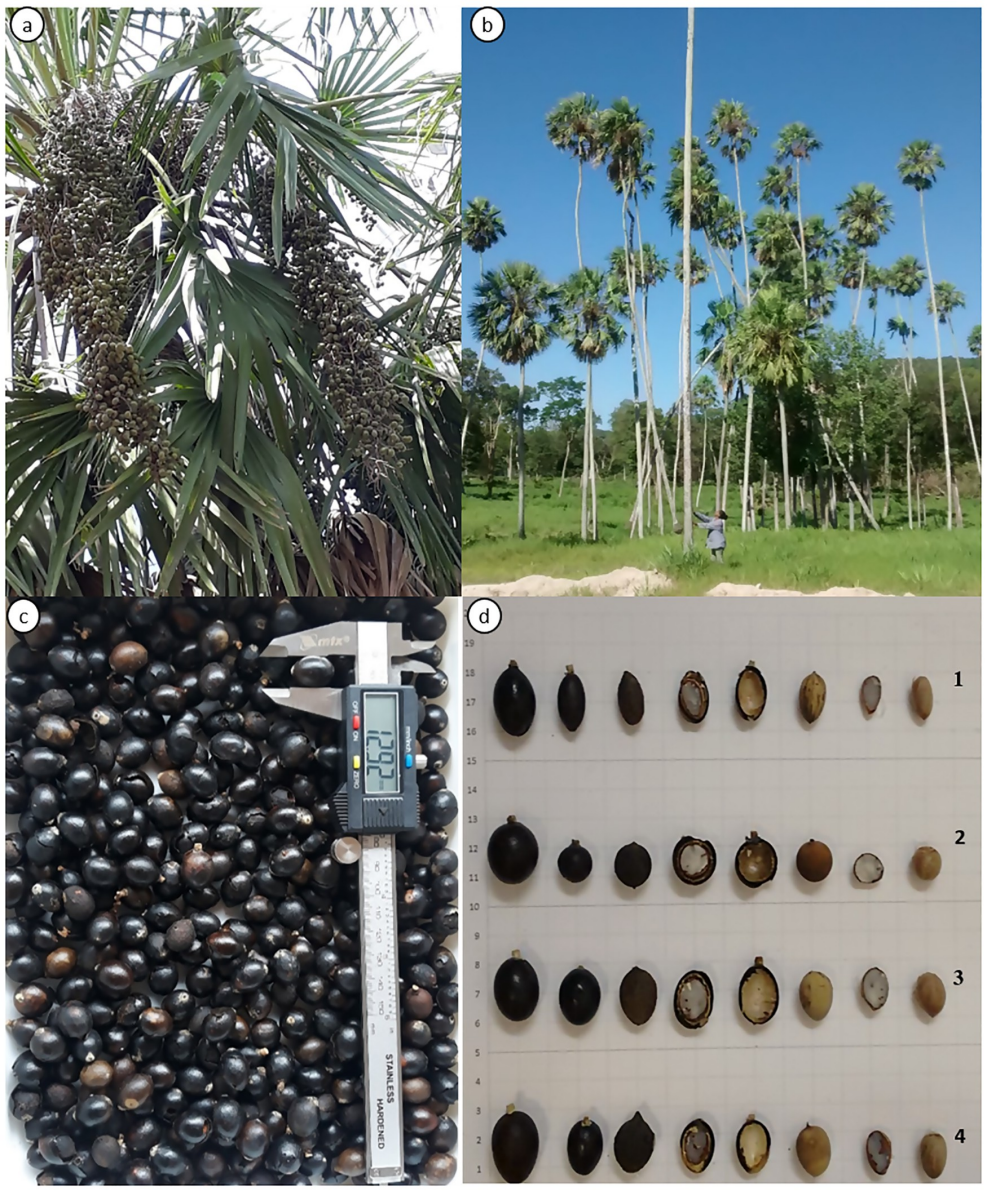
*Copernicia alba* Morong ex Morong & Britton (Arecaceae, caranda): Plant habit, fruit and seeds morphology. a. plant habit in a carandazal formation; b. branches with unripe fruits; c. Ripe fruits; d. Ripe fruits and variations in seed morphology (1- fusiform; type 2- spherical; type 3- ellipsoid; type 4- ovoid; 1^th.^ to 4^th^ columns. Fruit after removal of pericarp (5^th^ column). Seed exposed after extraction of seed coat (6^th^ to 8^th^ column).

Despite forming extensive populations in the Pantanal, little is known about the morphology, anatomy, and chemical composition of the *Copernica alba* fruit used as food by the local fauna and people who live in the surroundings of the carandazais. In Pantanal populations of *carandá*, we verified that ripe fruits could vary in pericarp color (brown, black, or slightly orange) or shape (fusiform, spherical, ellipsoid, or ovoid (Fig 1).

Considering the productivity of the fruits and their potential use in the food and pharmaceutical industries, our objective was to verify if there are morphological, anatomical and chemical differences in the different types of fruits found in wild populations of caranda in the Brazilian Pantanal, as a subsidy to the management of natural populations and as a basis for large-scale commercial plantations.

## Material and methods

### Study area and botanical material

For anatomical and morphological characterization, completely developed fruits of *Copernicia alba* Morong ex Morong & Britton were collected from 30 individuals in Moenda Farm owner, Pantanal of the Paraguay, in Corumbá, MS [10] Coordenates 19°25’47.18"S e 57°53’33.13"W. Fertile specimens (collector number R.H. Silva 1.251) were herborized according to standard procedures and deposited in the Herbarium CGMS, UFMS.

Collection and transport authorization for the study was provided by the Ministry of Environment (MMA/SISBIO).

### Fruits: Morphometric analyses and yield

For morphological analyses of fruits and seeds, we collected 50 ripe fruits from five plants. We evaluated shape, color, texture, dimensions (length and diameter) and weight. The seeds were evaluated for texture, dimensions (length and diameter), fresh weight and dry weight. Dry weight was obtained on an electronic precision scale from fruit samples after drying in an oven at 105ºC until constant weight. We measured fruit dimensions with a digital pachymeter.

Initially, we determined if fruits and seeds presented significant morphological variations. This way, a set of morphological variables was summarized by Principal Component Analysis (PCA, function prcomp, package stats [16], 2021): fruit length and diameter, exocarp, mesocarp and endocarp thickness, endosperm diameter and length, exocarp, mesocarp, endocarp, endosperm fresh mass, exocarp, mesocarp and endocarp dry mass, and endosperm dry mass. Since the variables are on different scales, we opted to use the matrix of association by correlation. The two first axes of PCA represented approximately 61% of the total variation of data, and they were utilized to construct a graph to represent the distribution of samples (individuals of *C*. *alba*) in the multivariate space (function ggplot, package ggplot2, [17]).

To determine differences in fruit yield, specified by fresh mass of exocarp, mesocarp, endocarp, and endosperm, we built Generalized Linear Model with distribution of errors Gamma (GLM, function glm, package stats, R Core Team, 2021). The statistical significance test was calculated by analysis of squared deviations of the type II (function Anova, package car, [16].

We built general linear models (GLM, function glm, package stats [16], to determine differences in diameter, infolding number and depth of *C*. *alba* seeds according to four fruit types. The model was first tested with the Poisson distribution of errors for the variables number and diameter of seed infoldings for being discrete data. To verify if overdispersion occurred, we calculated the division of the residual standard deviation of the model by the degrees of freedom. We maintained the model with the Poisson distribution of errors, as we did not detect overdispersion. For the variable seed infolding depth, the model was built with the Gamma distribution of errors for being continuous data. The significance test for the three models was calculated by analyzing square deviations type II (function Anova, package car, [18]). When a significant mean difference was detected in the GLM, we applied the Tukey test to determine in which factor level combinations the differences were observed (function glht, package multcomp, [19]).

### Identification of the xylan in *Copernicia alba* seeds

The properly macerated seed (1g) was incubated with 1 mL of McIlvaine buffer, pH 5.0 [20] and 1 mL of purified *Aspergillus japonicus* xylanase [21]. The mixture was incubated at 50°C for up to 2 hours. The reaction was interrupted with 3,5-dinitrosalicylic acid (DNS-Merck, DEU) and the absorbance of the supernatant was determined at 540 nm using Genesys 10S UV-VIS spectrophotometer (Thermo Scientific, USA). The amount of released reducing sugars was quantified by the methodology according to [18] using a standard curve of xylose. One unit of activity (U) was defined as the amount of enzyme capable of releasing 1 μmol of reducing sugars per minute. Assay control was performed using 1% (w/v) birchwood xylan (Sigma-Aldrich, USA) as substrate under the same conditions described above.

### Hydrolysis products analysis in thin layer chromatography (TLC)

Xylanase activity was verified by assaying the activity against 1% (w/v) birchwood xylan (Sigma-Aldrich) and *Copernicia alba* seed as described in the item above. To assess the presence of xylan in *Copernicia alba* seed, we utilized the purified enzyme endo-xylanase of *A*. *japonicus*. We used 1% (w/v) of birchwood xylan as a control. The sample was centrifuged at 5.000g for 10 minutes, and the supernatant was used hydrolysis products on Thin layer chromatography (TLC) TLC was performed using a solvent system containing ethyl acetate, acetic acid, formic acid, water (9:3:1:4 per vol). The compounds formed were detected by spraying the TLC with a solution of H2SO4 and methanol (1:9 per vol.) that contained 0.2% orcinol, followed by heating at 100°C.

### Anatomical aspects

After collecting, the fruits was fixed in 10% buffered neutral formalin for 48 hours, washed with water, dehydrated in a crescent series from ethanol/butyl acetate to 100% butyl acetate, embedded in histological paraffin and sectioned on a 12 μm, using a rotary microtome (Leica RM-2145; Leica Microsystem, Germany). Paraffin was removed with 100% butyl acetate. Then, the slides were progressively hydrated in ethanol/water series, stained in safranin/alcian blue [22], again dehydrated in ethanol/tertiary butanol series, and mounted in synthetic resin (Entellan). Aiming to complete anatomical analysis parts of fruits was cut in appropriate pieces and embedded in glycolmethacrylate (Historesin^®^), cross sectioned (8 μm thickness) in a rotary microtome (Leica RM 2155), and stained with Toluidine Blue 1%. All slides were analyzed under a light microscope (Olympus CX-41F). Photomicrographs were obtained with the aid of microscope (*Leica* DMLB, Leica Microsystem, Germany) coupled to the image capture system and camera (*Leica* DC 300F, Leica Suite Application v.3.0.8 software).

The histochemical tests were performed from cross-sections on fruits/seeds using the following methods: Sudan IV and Sudan Black for total lipids [23] Nile blue for acid and neutral lipids [24], ferric chloride for phenolics [25], dissolution in hydrochloric acid was used for identify calcium oxalate crystals [26]), phloroglucinol hydrochloride to identify lignified cell walls (Johansen, 1940), Blue bromophenol was used to identify proteins [27], Schiff/PAS reagent to identify neutral polysaccharides [28] and IKI to identify starch grains [25].

## Results and discussion

### Fruits: Morphometric analyses and yield

The study indicated that the fruits of *Copernicia alba* are morphologically different in terms of length and width, confirming the different types recognized here. Our results differ from studies that report the predominance of a spherical shape for the species. According to [2], *Copernicia alba* presents fruits predominantly spherical though showing slight shape variations (ellipsoid, fusiform, and ovoid). The spherical shape is known as the pattern for the Coryphoideae subfamily. The variations in shape can be derived from intrinsic factors (position of fruit on the plant or of the seed on the bunch) or related to the environment.

**Fig 2:**
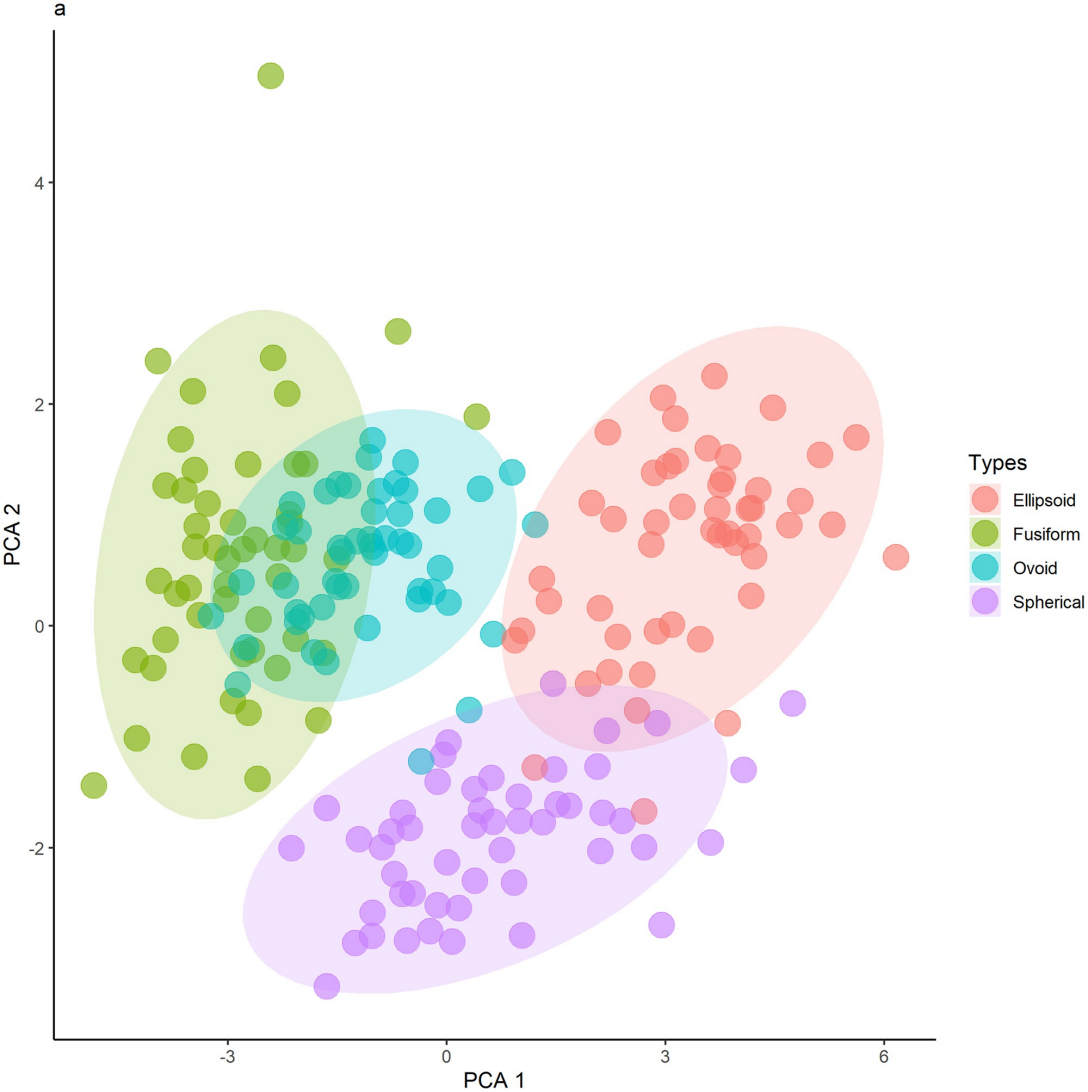
Biplot with the dimensions 1 and 2 of PCA by correlation capturing 60.8% of the original variability. The ellipses show a separation in the multivariate space of different *Copernicia alba* phenotypes concerning morphometric and mass variables.

The sampled fruits and seeds of *C*. *alba* showed morphological variations and were grouped into different categories; type 1, with elongated shape, named fusiform; type 2, spherical; type 3, or ellipsoid; and type 4 or ovoid (Fig 1). The four types are different regarding morphometry and weight, represented by the PCA orthogonal axes (MANOVA: Pillai-Trace = 1.55, F3,196 = 224.10, P<0.001, Fig 2, S1 Table). The type 1 fruit is more associated with thickness and length, and type 4 with diameter and weight, confirming the variation in shapes, sizes and weights of fruits found in the assessed *C*. *alba* populations. Our results probably indicate that these differences are from genetic origin once the fruits were collected in monodominant formations submitted to similar environmental conditions. There is also correspondence between these features and other morphologic characters of the individuals.

Regarding total yield in the evaluated samples, the exocarp represented 19.54% of the fruit, the mesocarp 17.39%, the endocarp 10.36% and the endosperm 52.70%. About types, F1 and F4 were the most discrepant, with the lowest and the highest proportion of exocarp, respectively; types F2 and F3 always had similar values.

**Fig 3:**
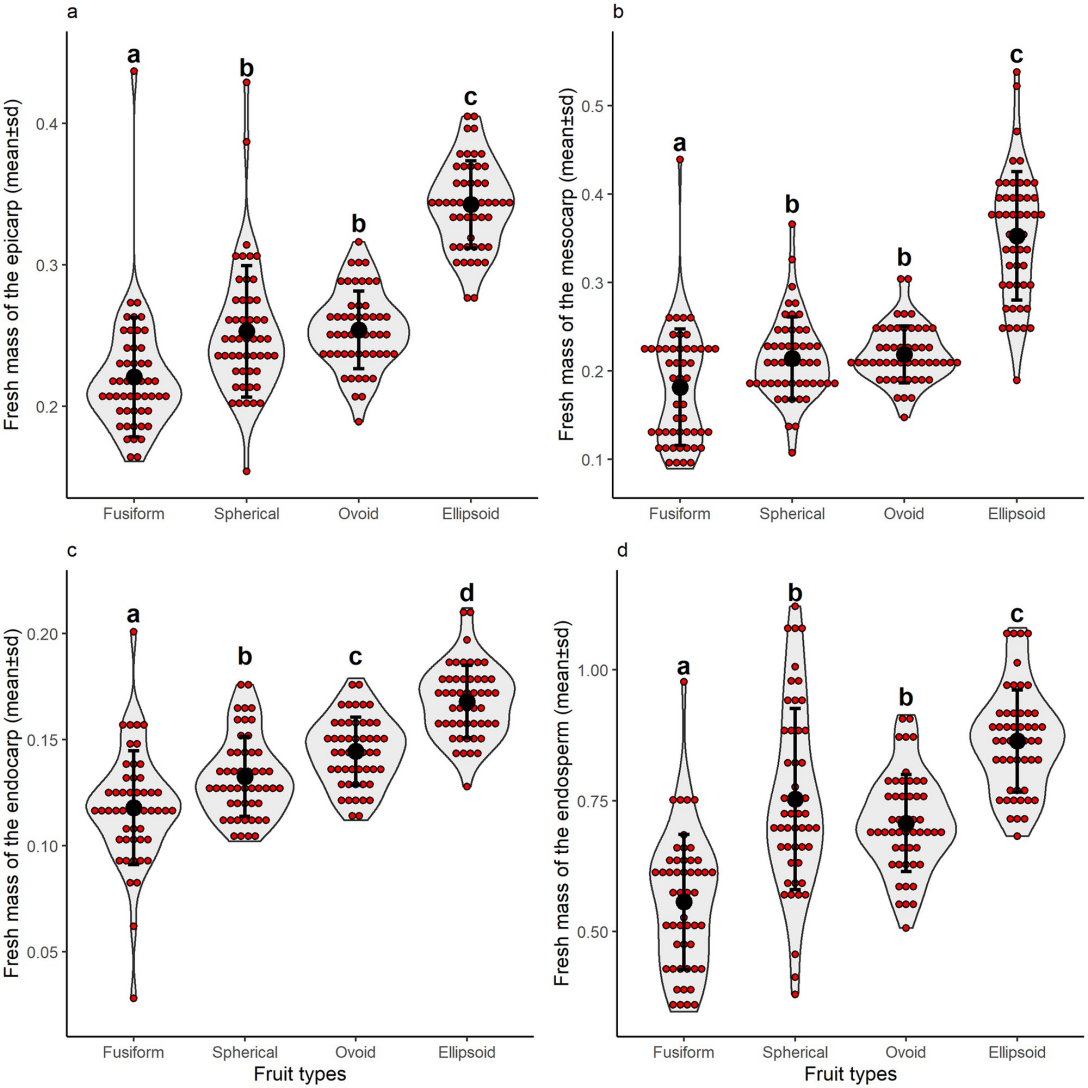
Yield of fresh masses of (a) exocarp, (b) mesocarp, (c) endocarp, and (d) endosperm of *Copernicia alba* fruits concerning phenotypes. Red dots after the mean and standard deviation represent the empirical data. The gray area is the density of the points distribution. Different letters for factor levels represent significant mean difference under Tukey’s test.

Concerning yield, we observed a sharp variation between phenotypes (Exocarp: Gamma GLM, χ^2^ = 237.96, P≤0.001, Fig 3a; Mesocarp: Gamma GLM, χ^2^ = 214.72, P≤0.001, Fig 3b; Endocarp: Gamma GLM, χ^2^ = 140.29, P≤0.001, Fig 3c; Endosperm: Gamma GLM, χ^2^ = 144.31, P≤0.001, Fig 3d), as well as between fruit parts. The differences allow for applications of fruit types; thus, we suggest the types F1, F2 and F3 for industrial processing of products with higher added value, such as oil, cosmetics and creams. In contrast, the F4-type fruit would be best indicated for pulp extraction for its higher mesocarp yield. Various reports evidence the success of pulp extracted from native fruits, e.g. *Euterpe oleracea* (*açaí*, in Portuguese), in Amazonia, utilized as food complement consumed as juice, flour, mousse with fish, ice creams, creams, jellies and cakes, which became commercially famous. Our results of pulp yield suggest that *C*. *alba* fruits have potential use for food since the pericarp has characteristics comparable to those of *açaí* with a pulp yield of 17% [26].

**Fig 4:**
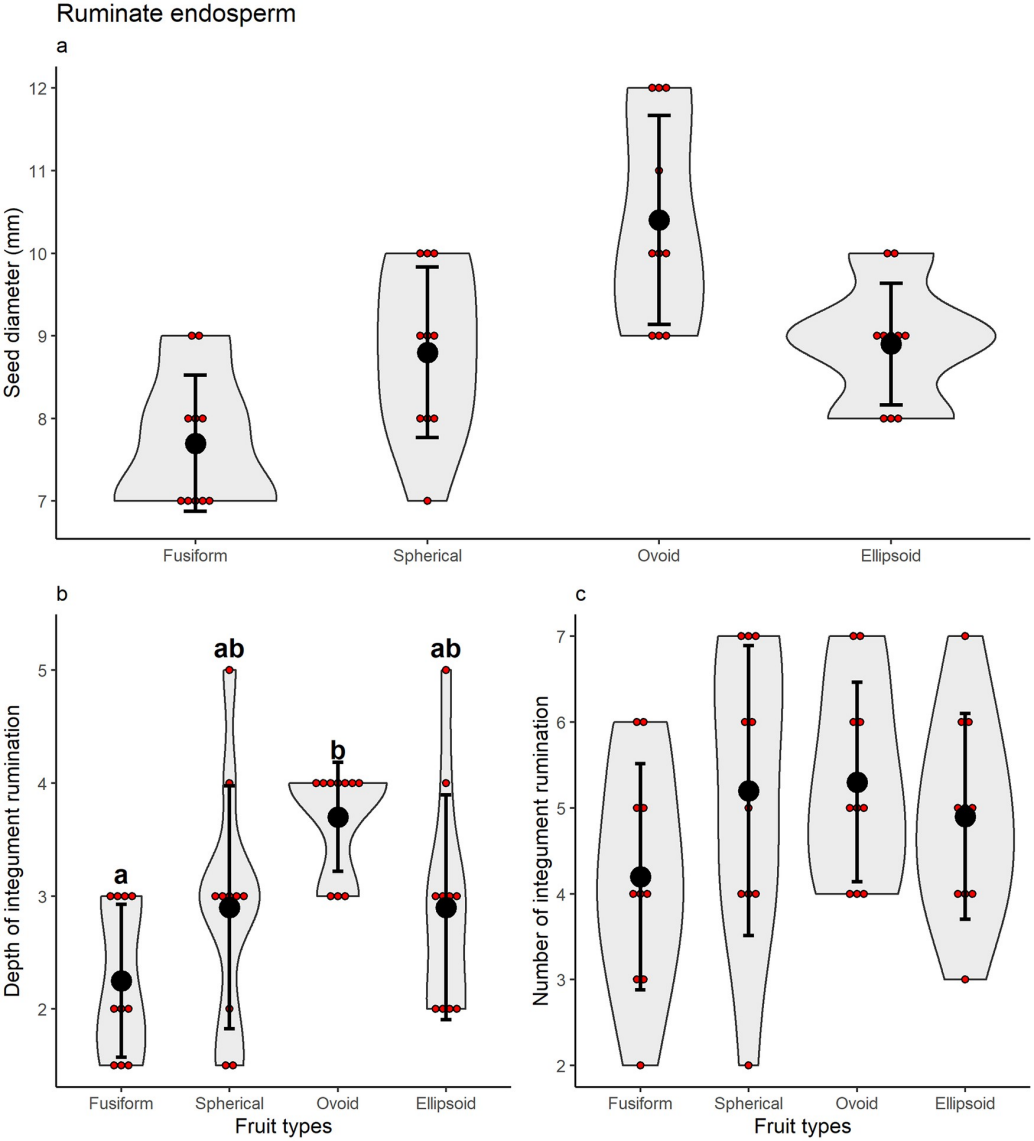
Differences in (a) diameter, and (b) infolding depth and (c) number in ruminate seeds of *Copernicia alba* (Arecaceae) by function of different fruit types. Red dots after the mean and standard deviation represent the empirical data. The gray area is the density of the points distribution. Different letters for factor levels represent significant mean difference under Tukey’s test.

Seed diameter did not vary between fruit types of *C*. *alba* (GLM Poisson: χ^2^ = 4.09, P = 0.252, Fig 4a) nor the number of seeds infoldings (GLM Poison: χ^2^ = 1.55, P = 0.671, Fig 4c). Seed infolding depth varied among the four different *C*. *alba* fruit types (GLM Poisson: χ^2^ = 13.58, P = 0.004, Fig 4b). The fusiform type differed from the ovoid type (z = -3.517, P = 0.002, Fig 4b) and was similar to the spheric (z = -1.860, P = 0.243, Fig 4b) and ellipsoid types (z = -1.860, P = 0.243, Fig 4b). The ovoid type was similar to the spheric (z = -1.787, P = 0.276, Fig 4b) and ellipsoid types (z = 1.787, P = 0.276, Fig 4b).

### Identification of the xylan in *Copernicia alba* seeds

**Fig 5:**
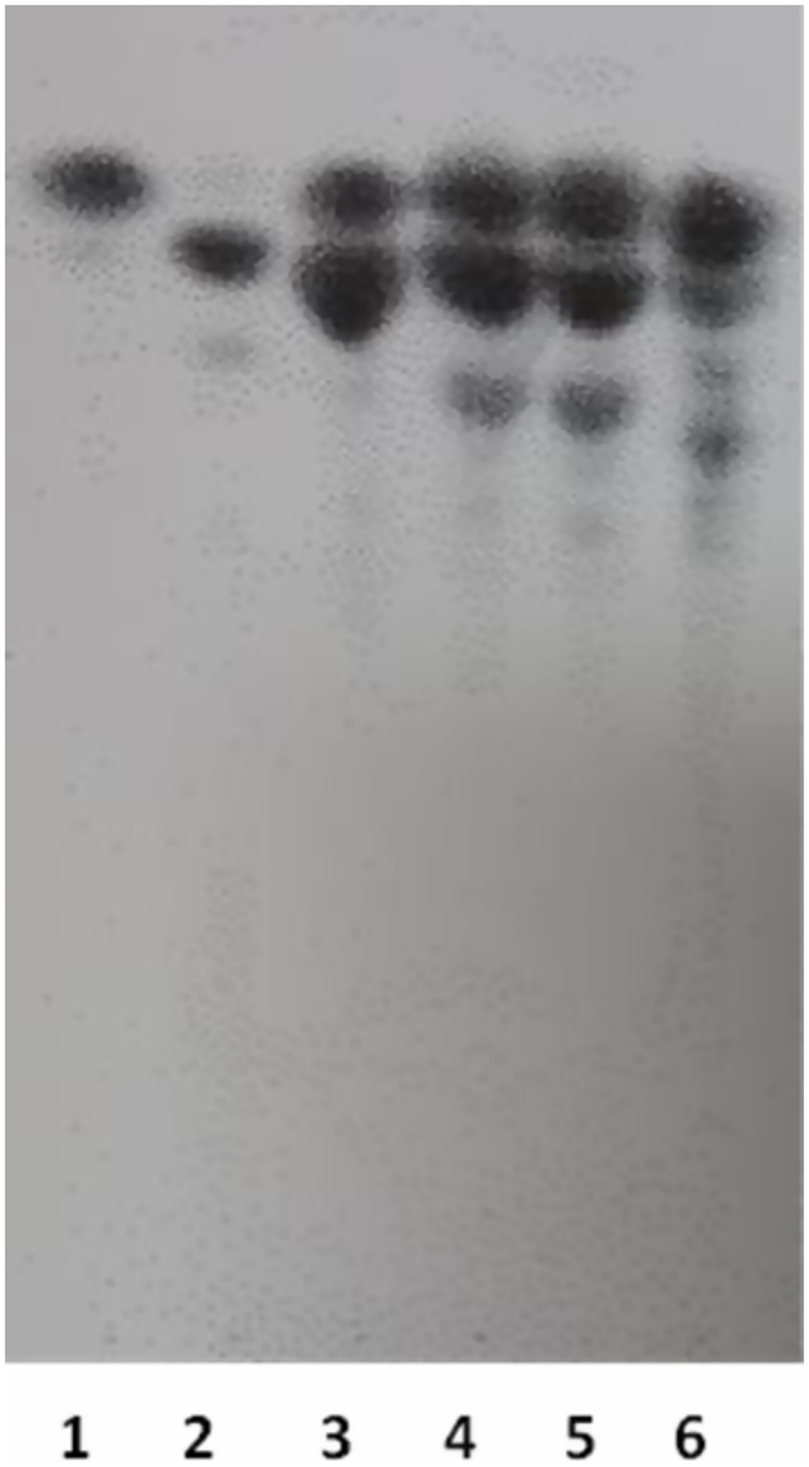
Thin-layer chromatography (TLC) of hydrolysis products using 1% (w/ v) of *Copernicia alba* seed and birchwood xylan substrate upon reaction with endo-xylanase from A. japonicus. Line 1: xylose 1 mg/mL; Line 2: xylobiose 1 mg/mL, Lines 3–4: hydrolysis products using xylanase after 30 and 60 min of incubation, using Copernicia alba seed, Lines 5–6: hydrolysis products using xylanase after 30 and 60 min of incubation, using birchwood xylan.

The purified endo-xylanase of *A*. *japonicus* showed high specificity for both tested xylans, demonstrating 100% of activity for birchwood xylan and 87% of activity against xylan of *Copernicia alba* seed (Fig 5). Like other endo-xylanases [29], the xylanase of *A*. *japonicas* [21] is specific to breaking glucosides β-anomeric of xylose residues. Purified xylanases of *A*. *flavus* and *A*. *terreus* had also been tested against various substrates (avicel, starch or carboxymethylcellulose, xylan), showing activities only for xylan [30, 31].

The hydrolysis products of xylan of *Copernicia alba* seed and birchwood xylan by purified endo-xylanase of *A*. *japonicus* were analyzed through Thin Layer Chromatography (TLC). The results demonstrated the formation of xylooligosaccharides of various lengths and xylose for both xylans of *Copernicia alba* seed and birchwood (Fig 5). The endo-xylanases randomly cleave the xylan chain of the β-1,4-glucosidic bonds and liberate xylooligosaccharides of various lengths. Our result corroborates those described by [21]. Due to the high selectivity of the xylanases, and based on the results found in the enzymatic test and TLC, it is possible to state that *Copernicia alba* seed presents a high content of xylan, with potential for use in several sectors, such as in the food industry, cosmetics, medicines, and in the production of second-generation bioethanol [12, 15].

### Anatomical aspects of fruits and seeds

**Fig 6:**
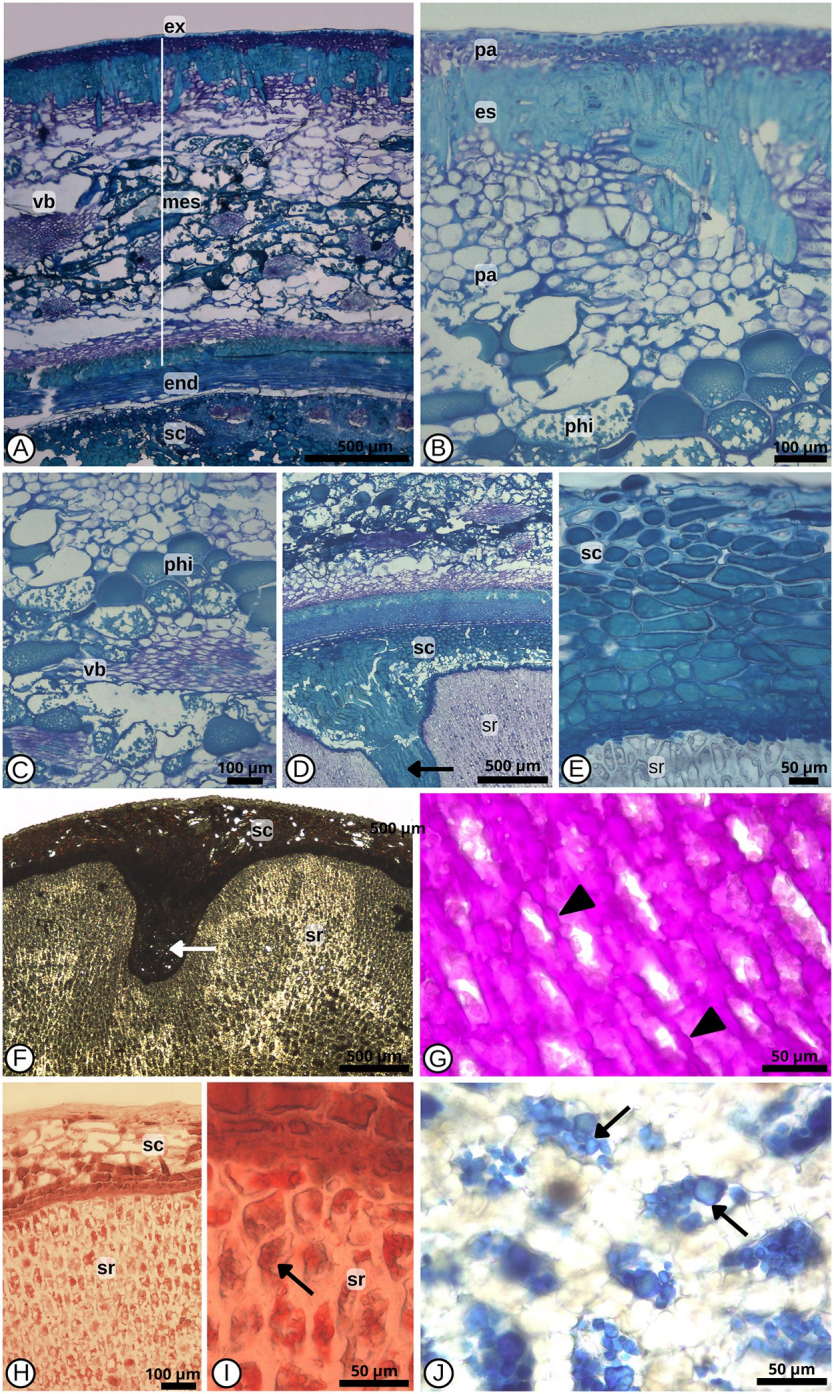
General anatomical features of *Copernicia alba* (Arecaceae) mature fruit and seed in cross section. (A) General view showing exocarp (ex), mesocarp (mes) with parenchyma and esclerechyma layers interspersed, endocarp (end) and vascular bundle (vb), and seed coat (sd) (mep). (B) Detail of pericarp: parenchyma (pa) and sclerenchyma (es) layers. In the parechyma tissue observe the phenolic idioblasts (phi). (C) Detail of parenchyma internal part with phenolic idioblast (phi) and vascular bundle (vb). (D) Internal layer of pericarp and part of seed coat (sc) structure: observe the seed coat forming projection (arrow) into seed reserve (sd). E. Detail of multilayered seed coat (sc) and cells of seed reserve (sr). Histochemical reactions in seed coat (sc)and reserve endosperm (sr): (F) Ferric chloride to identify phenolic compounds (white arrow); (G) Thick primary cell walls stained pink after reaction with Schiff/PAS reagent to identify neutral polysaccharides (arrow heads); (H-I) Cell content after reaction with Sudan IV showing lipidic granules in orange (arrow); (J) Cell content after reaction with Blue bromophenol to identifying proteins (arrows).

Several Arecaceae provide a high quantity of fruits, from the colored to the dark, for frugivore animals in the tropics [32]. The ripe fruits of *C*. *alba* are covered by a thick, rigid but brittle exocarp. The fruit cuticle, smooth and shiny, and its small size can be considered attractive to several animals, such as coatimundi, monkeys, tapir, rodents, birds, and fish [2], contributing effectively to the species dispersal over the Pantanal plain. Palm fruits can vary widely in the consistency and composition of the pericarp, from fleshy to fibrous and with big cavities that allow buoyancy, as in *Cocos nucifera* L. (Arecoideae). Fruits of the single-seeded berry type, as observed in *C*. *alba*, are considered less frequent in the Arecaceae family, where fleshy pericarp of the drupe type predominate [33]. The pericarp is thin similar to that observed in *Oenocarpus minor* Mart. and *Euterpe oleraceae* Mart. [34, 35]. The exocarp of *Copernicia alb* a fruit is uniseriate and formed by cells with thickened walls, covered by a thick cuticle and wax layer (Fig 6A and 6B). Uniseriate exocarp covered by a thick cuticle was detected in other palm fruits belonging to the Borasseae clade, Coryphoydeae [36]. Immediately under the exocarp, we identified the mesocarp, composed by parenchyma end sclerenchyma layers, interspersed (Fig 6A). Near the exocarp, there are five layers of parenchymatic cells with primary thickened walls full of phenolic compounds (Fig 6B). The phenolic compounds give the typical dark color to the mature fruits. Besides attracting local fauna, fruit colors can also indicate possible successional stages of the plant community, as suggested by the studies of [37], that black fruits are significantly related to pioneer species.

Under the parenchyma layers, we observed the sclerenchyma composed of long sclereids with thickened, strongly lignified walls, and with reduced lumen (Fig 6B). These cells are elongated, and are organized standing up forming a palisade in relation to other tissues. Internally to this fibrous layer the inner is formed by parenchymatic cells with soft walls, a slightly sweet tissue, slightly thickened, not lignified (Fig 6B and 6C). In this part of the mesocarp we observed numerous idioblasts with phenolic compounds. The substantial presence of phenolic compounds in palm fruits can have a relevant role in protecting fruits and seeds against high temperatures ordinary in areas of occurrence of *C*. *alba* [37], as well as against fungi and bacteria, due to antimicrobial activity [38, 39]. Several palms have been investigated as potential sources of bioactive compounds that can be obtained from roots, flowers, leaves and fruits, with different biological activities, such as antioxidant, antimicrobial, analgesic, antipyretic and antiinflammatory, antimalarial and antibacterial, being most of those pharmacological properties reported in ethnobotanical studies proved in scientific research [40]. Therefore, phenolic compounds and abundant cellulose identified with histochemical tests in seeds of *C*. *alba* could be explored investigated as molecules useful to human health, being an option to meet the demand of the antioxidant in the food industry.

The vascular system of fruit is formed by collateral vascular bundles associated with large fibrous caps present in the mesocarp (Fig 6A and 6D). Fibers associated or not with the vascular system are a marked trait of Arecaceae and represent a relevant taxonomic attribute at a generic or specific level [41, 42]. In this region mesocarp we observed idioblasts containing crystals of the raphide type. Raphides are needle-shaped crystals of calcium oxalate insoluble present throughout the Monocotyledons, and ubiquitous in palms, found in roots, stems, leaves, flowers, and fruits. Calcium oxalate appears in plants is considered a protection against herbivory or insect attacks or even as calcium´s accumulation system, as all plants without calcium are unable to grow or to complete their life cycles [43]. Insoluble crystals produced by plants has been proposed as a defense mechanism against herbivore insect or other little animals when they chew on these plants. The crystals produce minor injuries in the mucosa inducing severe irritation and damage herbivores since crystals containing enzymes with antitrypsin activity stimulate the release of histamine and bradykinin resulting in puncture and lesion the cellular structures accompanied by the release of vasodilating agents that lead to edema and congestion [44]. The endocarp is composed by layers of sclerenchyma cells rows of sclerified cells arranged perpendicularly to the seed axis, forming a thick coat (Fig 6A and 6D).

Internally, the single seed is covered by a complex multilayered structure forming a relatively thin seed coat, papyraceous, darkened that forms projections inside of the reserve tissue (Fig 6D and 6E), so producing an endosperm of the ruminated type (Fig 6F), a pattern observed in other palm species [41]. *Copernicia alba* seeds are ruminated with irregular folds, due to folds or invaginations of the seed coat and have different present different levels of penetration in the endospermic tissue among the evaluated types. The ruminated seed is reported to the family Arecaceae by [35, 36, 45]. In *Euterpe oleraceae* seeds these projections have thin walls, are pectocellulosic and rich in reserve substances [35]. The function of this structure in seeds has not yet been defined. According to [46], endosperm rumination, by causing an increase in the contact surface between the seed coat and endosperm, may facilitate the entry of water, oxygen and/or nutrients to embryo during germination. Fruit ontogeny is suggested to clarify this function. The internal layers of the seed coat tissue present thickened and lignified walls and contain abundant phenolic compounds (Fig 6D–6E). The presence of phenolic idioblasts in the seed coat layer provides an efficient protective barrier against predator attack due the chemical properties of phenols and considering the demand of a diversity of organisms for the seed nutritional reserves [47]. Phenolic compounds can protect seeds with deterrent potential against many insects, as shown experimentally [48].

Under the seed coat, we observed the endosperm, the primary reserve tissue, and the embryo (Fig 6F and 6G). The endosperm cells are parenchymatic and present primary cell walls, strongly thickened, with numerous pit fields interlinking the whole tissue. Inside the endosperm cells, we observed a sharp reaction indicating the presence of lipids and proteins and the absence of starch, independently of fruit type, shape or color (Fig 6H–6J). In endosperm tissue, cells have cellular primary cell walls, despite very hardened, they are not lignified, composed of several layers of reserve polysaccharides. Preliminary evaluations indicate that the endosperm cell walls contain hemicelluloses of the type xylans. Together with other polysaccharides, proteins and lignin, xylans constitute the most abundant biopolymers on Earth and are crucial for plant structure [49]. The xylooligosaccharides extracted from xylans can come from several sources, such as wheat bran and straw, garlic straw, sugarcane bagasse, peanut shell, among others [50]. Xylans have great applicability in producing bandages as a function of their antimicrobial activities and as functional food given their gut prebiotic property [50, 51]. Functional features and chemical composition of the hemicelluloses found in the endosperm of *C*. *alba* seed are yet unknown; they need to be investigated to identify potential functional properties mentioned for these carbohydrates in other plants.

**Fig 7:**
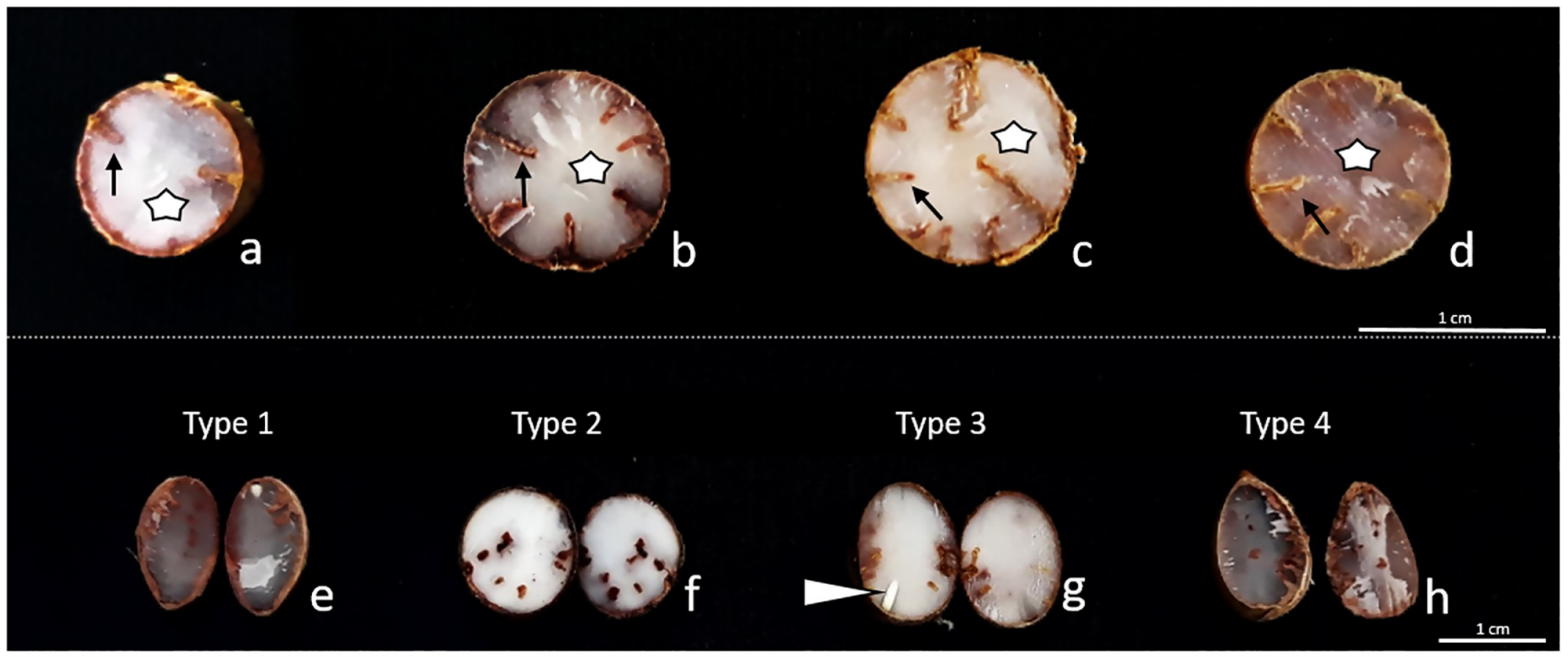
Seeds of *Copernicia alba* (Arecaceae) in cross section (a-d) showing tegument with infoldings (arrow) and endosperm (white star) in each type. Seeds in longitudinal section (e-h): tegument, endosperm and embryo (arrow head). Scale bars: 1 cm.

In *C*. *alba* the embryo is composed is short and straight axis (nearly 2–3 mm high and 1 mm wide), little developed (Fig 7g). Attributes such as position, size and shape of embryos vary widely in Arecaceae, from the big dimensions as in *Oenocarpus bacaba* (with 13 mm diameter) to small ones as in *Euterpe* species, with 2 mm in diameter [33].

The bibliographic survey indicated the scarcity of information on the anatomy of the fruit and seed of *C*. *alba*, thus pointing to the urgent need for anatomical and ontogenetic studies of this species to improve a better genetic and economic use. Thus, the preliminary characterization presented here was elaborated based on existing studies on the anatomy of the fruit and seed of other palm trees.

## Conclusions

As for the anatomical characteristics, the fruit has a shiny pericarp and contains phenolic compounds in the dark mesocarp, which is possibly related to the attraction of the fauna that feeds and disperses the seeds, in addition to providing protection against variations in temperature and radiation, protecting the embryo. As a reserve, the seed has high content of xylan, indicating bioenergetic and biotechnological potential. Quantitative and qualitative data on the economic use of fruits and seeds and endosperm can point to possibilities of sustainable economic use fruits and seeds and endosperm can point to possibilities of sustainable economic use of *C*. *alba* fruits and seeds as a source of bioactive substances and sugars in the production of functional foods.

## Supporting information

S1 Table Correlations between the 14 morphometric and mass variables of *Copernicia* alba phenotypes with two ordering axes produced by principal component analysis. In bold, the strongest correlation values between the variables that contributed most to the formation of the axis.
(XLSX)
